# A Comparative Study of Transbuccal and Extraoral Approaches in the Management of Mandibular Angle Fractures: A Systematic Review

**DOI:** 10.3889/oamjms.2016.096

**Published:** 2016-09-02

**Authors:** Sabah Ali Beza, Sayed Attia, Edward Ellis, Layla Omara

**Affiliations:** 1 *Department of Oral and Maxillofacial Surgery, Faculty of Oral and Dental Medicine, Cairo University, Cairo, Egypt*; 2 *Department of Oral and Maxillofacial Surgery University of Texas Health Science Center at San Antonio, TX, USA*

**Keywords:** extraoral approach, transbuccal approach, mandibular angle fracture, trocar canula, internal fixation, postoperative complications

## Abstract

**AIM:**

The aim of the present study was to compare the extraoral and transbuccal approaches for the treatment of mandibular angle fractures with regard to postoperative complications.

**PATIENTS AND METHODS:**

An electronic search for relevant articles without language and date restrictions was performed in July 2016. Inclusion criteria were studies in humans including randomised controlled trials (RCTs), controlled clinical trials (CCTs), prospective studies (PS), and retrospective studies (RS). In total, 107 patients were included from four studies (transbuccal = 48, extraoral = 59). The follow-up period varied from 3 months to 24 months.

**RESULTS:**

In extraoral group the average of unsightly scar, facial nerve weakness, infection, malocclusion, plate removal were found to be 55% (range, 10% -100%), 26.5% (range, 0%-53%), 11.7% (range, 0% - 20%), 22.5% (range, 0% -50%), 6.7% (range, 3.3% - 10%) respectively while these parameters in the transbuccal approach were found to be no obvious unsightly scar, 6.6 % (range, 0%-13.3%), 8.1% (range, 0% - 20%), 4.8% (range, 0% - 12.5%), 0%. The incidence of postoperative trismus and nonunion/malunion were 0% in both groups.

**CONCLUSION:**

The results of this study suggest that transbuccal approach shows fewer complications than extraoral approach when used for the treatment of mandibular angle fractures.

## Introduction

The objective of mandibular fracture treatment is the restoration of anatomical form and function, with particular care to establishing the pre-trauma occlusion. Traditionally, this has been achieved by immobilising the jaws using various dental wiring techniques. In the previous two decades, interest has increased for different methods of open reduction and internal fixation [1]. Methods of open reduction and internal fixation have continued to evolve and have changed enormously in the last few years with the advent of plate and screw fixation hardware. Fixation devices became smaller, simpler to handle, and extraoral incisions have been minimised. However, there still is debate regarding the optimal treatment [2, 3].

Mandibular angle fractures (MAFS) have a high frequency of complications particularly in relation to the insufficient stability of the fixation systems [4-6]. Despite the advances in internal fixation used for the treatment of fractures of the mandibular angle, these fractures still present unpredictable results and difficulties in treatment compared to other mandibular fractures. A large number of studies testifies to the fact that no single approach has been shown to be ideal [7].

Extraoral approaches were traditionally used for open reduction and internal fixation of mandibular angle fractures. It has the potential disadvantage of leaving an unaesthetic scar and risks damage to the facial nerve, though the advantages are better exposure and direct application of plate fixation [8-10]. The transbuccal approach has the advantages of no external scarring and direct visualisation of the occlusion during placement of the bone plates injury to branches of the facial and other anatomic structures were reduced [9-12].

In the previous decades, increased availability of high quality and easy-to- use trocar instrumentation has made the transbuccal approach prevalent, but research into its complication rate is greatly lacking. Presently, the choice of the approach relies on the surgeon’s personal preference [13].

The aim of this study is to focus on the question: “Is there a significant difference in the clinical outcomes between the transbuccal versus extraoral approaches in the management of mandibular angle fractures?

## Patients and Methods

### Data sources and keywords

An electronic search was performed without language and date restrictions in July 2016 in the following data databases: Pub Med, Cochrane Database of Systematic Reviews, Cochrane Central Register of Controlled Trials (CENTRAL), Alt Health Watch, Health Source: Consumer Edition, Health Source: Nursing/Academic Edition, Scopus, Wily Online Library, and Electronic Journal Centre.

The keywords and their combinations used in this search included:

1. In PubMed: ((extraoral[All Fields] AND approach[All Fields]) OR(extroral[All Fields] AND technique[All Fields])) AND (transbuccal[All Fields] AND approach[All Fields]) OR ((“mandible”[MeSH Terms] OR “mandible”[All Fields] OR “mandibular”[All Fields]) AND angle[All Fields] AND (“fractures, bone”[MeSH Terms] OR (“fractures”[All Fields] AND “bone”[All Fields]) OR “bone fractures” [All Fields] OR “fracture” [All Fields]))(700 articles) were collected from this database.
2. In Scopus: “extraoral approach” or” extraoral technique “or” transbuccal approach” and “mandibular angle fracture”(174 articles) in all years.
3. In Wily Online Library: extraoral approach or extraoral technique (in Full Text) OR transbuccal approach in Full Text AND mandibular angle fracture (in Full Text) (195 articles). A manual search of oral and maxillofacial surgery related journals including British Journal of Oral and Maxillofacial Surgery, the International Journal of Oral and Maxillofacial Surgery, Journal of Maxillofacial and Oral Surgery, Journal of Craniofacial Surgery, Journal of Oral and Maxillofacial Surgery, Oral Surgery, Oral Medicine, Oral Pathology, Oral Radiology and Journal of Cranio-Maxillofacial Surgery was performed. Relevant reviews on the subject and the reference lists of the studies identified were scanned for possible additional studies.

### Inclusion and exclusion criteria

Inclusion criteria were studies in humans including randomized controlled trials (RCTs), controlled clinical trials (CCTs), prospective studies (RS), retrospective studies (RS), unilateral or bilateral fractures of mandibular angle fracture with the aim of a comparative study between the extraoral approach and transbuccal approach with the use of transbuccal instrumentation for treatment of mandibular angle fractures with regard to postoperative complications and other factors. Exclusion criteria were: combined symphyseal and condylar fractures, comminuted fractures, edentulous patients, technical reports, case reports, in vitro studies, animal studies, and review papers.

### Selection of relevant studies

The following data were extracted from the studies included in the final analysis: authors, year of publication, study design, number of participants, patient age range and/or mean age, follow-up period, site of MFs, MAF, fixation methods, surgical approach, duration of operation, postoperative maxillomandibular fixation (MMF), use of antibiotics and/or chlorhexidine, teeth retained and removed in MFs, and postoperative complications including evaluation of the resulting scar from an aesthetic point of view, facial nerve damage evaluation, treatment of tooth in the fracture line and its implication on malunion and non-union, infection, postoperative malocclusion, need for plate removal and mouth opening.

### Assessment of Quality

A methodological quality analysis was performed by merging the proposed criteria of the Strobe statement [14], Moose statement [15], and Prisma statement [16], to verify the force of scientific evidence in making clinical decisions. The classification of the risk of potential bias for every article was based on the following criteria: random selection in the participants, the definition of inclusion and exclusion criteria, report of losses to follow-up, the validity of assessments, and statistical analysis. A study that comprised all the criteria mentioned above was categorised as having a low risk of bias, a study that did not comprise one of these criteria was categorised as having a moderate risk of bias. If two or more criteria were missed, the study was classified to have a high risk of bias.

## Results

**Figure 1:**
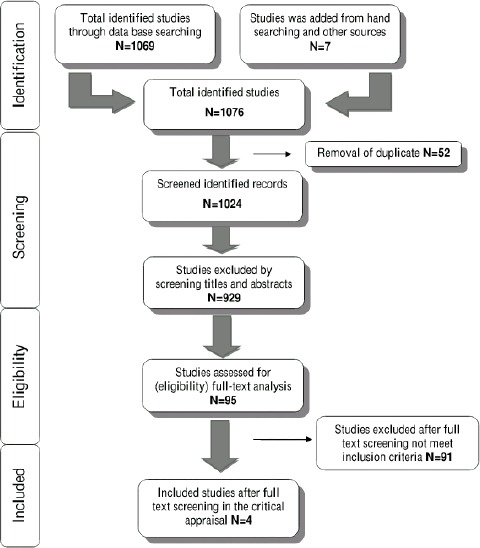
Flow diagram of study selection process

Summary of the study selection process is shown in Figure 1. The electronic search resulted in 1069 studies; seven additional articles were added from hand- searching and other sources. After the initial screening of articles, 52 articles were excluded because of duplication. Of the remaining 1024 articles assessed, 929 were excluded by title and abstract because they were not related to the topic. Ninety-five studies were selected for full-text analysis leading to the exclusion of 91 articles because they did not meet the inclusion and exclusion criteria. Thus, a total of 4 articles were included in the systematic review.

### Description of included studies

Extracted data of the included 4 studies are listed in Table 1. Three prospective studies [9, 10, 12], and one retrospective studies [17] were included in this study. A total of 170 patients were enrolled in the four studies, but 63 were excluded because they had other surgical approaches. This left a total of 107 patients with 48 patients in the transbuccal approach group and 59 patients in the extraoral approach group. The ages ranged from 16-62 years. The follow-up period varied from 3 months to 24 months. Additional MFs were reported in three studies [9, 10, 12]. Regarding the transbuccal approach group, three studies performed fixation using a single miniplate, in one of these articles performed the fixation on the lateral aspect of the mandible using 2 mm miniplate, a four-hole centrally spaced standard mini plate and 6-8 mm screws [17]. In the second article a single 2.5 mm, four whole stainless steel mini plate with a gap and 2.5 mm × 8 mm screws was placed along the lateral aspect of the mandible [9]. In a third article [10] used the same fixation method as the second study a single 2.5 mm non-compression, 4-holed with gap stainless steel mini plate and 6 or 8 mm monocortical screws was used. One of the four studies performed the fixation using 2 mini plates in that article [12], 2 mini plates were placed with approximately1 cm distance between them on the lateral cortex. The plate superiorly was a two-hole miniplate fixed superiorly on the external oblique ridge and the inferior plate was a four whole miniplate fixed along the lateral aspect of the mandible.

**Table 1:** Studies comparing management of mandibular angle fractures via transbuccal and extraoral approaches

Authors, Publication year	Study design	P (n)	Patient age range (mean), years	Follow up	Site of MFs	Methods of fixation MAF	Surgical approach	Duration of surgery, min, mean	Post operative MMF (n)	Antibiotics/ chlorhexidine, days	Teeth retained/ removed (in MAF)
Kale et al., 2010	PS	15	26.9y	7 day 2 weeks 3 months	Angle (n = 14), Body (n = 1), Para-symphysis (n = 8),	(G1) 2 mm two miniplates with around 1 cm distance on lateral cortex (n = 4) (G2) 2 mmt wo miniplates with around 1 cm distance on lateral cortex (n = 10)	Extraoral Intraoral + transbuccal	(G1) 63 (G2) 49.5	NP	NM	2/12
Kumar et al., 2011	RA	80	16-62 (26.6y)	weekly 3 months	Angle N = 80	(G1) four hole centrally spaced 2 mm, two miniplates (n = 30) (G2) four hole centrally spaced 2 mm, one miniplates (n =15) (G3) four hole centrally spaced 2 mm, one miniplates (n = 35)	Extraoral Intraoral + transbuccal Intraoral	NM	NP	NM	Retained teeth in line of fracture 73/NM
Patter et al., 2014	PS	30	NM	1 week 2 weeks 3 weeks 4 weeks 6 weeks 8 weeks 10 weeks 12 weeks 6-24 months	Angle fractures (N = 45) patients associated with other mandibular fractures	(G1) single non-compression 2.5 mm, 4 holed with gap stainless steel mini plate and 6/8 mm monocortical screws (n = 12). (G2) single non-compression 2.5 mm, 4 holed with gap stainless steel mini plate and 6/8 mm monocortical screws (n = 8). (G3) single non-compression 2.5 mm, 4 holed with gap stainless steel mini plate and 6/8 mm monocortical (screws (n = 10)	Intraoral approach Transbuccal approach Extraoral approach	NM	(G1)12 (G2)8 G3)10	NM	NM/1
Sudhakar et al., 2015	PS	45	16-51 (29.6y)	1 week 2 weeks 4 weeks 6 weeks 3 months 6 months	Angle fractures (N = 45) out of 45, 24 patients associated with other facial fractures	(G1) A single 2.5 mm, four hole stainless steel mini plate with gap and 2.5 mm × 8 mm screws, (n = 15) (G2) A single 2.5 mm, four hole stainless steel mini plate with gap and 2.5 mm × 8 mm Screws (n = 15) (G3) A single 2.5 mm, four hole stainless steel mini plate with gap and 2.5 mm × 8 mm screws	Intraoral transbuccal +intraoral approach extraoral approach	(G1) 65 ± 3.27 (G2) 93.5 ± 4.36 (G3) 85.5 ± 4.90	G1)15 (G2)15 (G3)15	Patients were admitted for IV antibiotics/NM	NM

P, participants; MAF, mandibular angle fracture; MMF, maxillomandibular fixation. MF, mandibular fracture; RA, retrospective analysis; PS, Prospective study; NM, not mentioned; NP, not performed.

Regarding the extra-oral group, there was one article [17] that used two 4-hole centrally spaced mini plates; one study [12] used two mini plates with approximately1 cm distance between them and the fixation performed on the lateral cortex; In one article a single 2.5 mm, four hole stainless steel mini plate with gap and 2.5 mm × 8 mm screws was placed on the lateral cortex [9]. In one article [10] a single non-compression 2.5 mm, 4-holed with gap stainless steel mini plate and 6 or 8 mm monocortical screws was used. Two of four studies [9, 12] provided information on the mean operation time.

### Assessment of Quality

The risk of bias outcomes is summarised in Table 2. Two [12, 17] were considered to be a high risk of bias and two were considered to be a low risk of bias [9, 10].

**Table 2:** Results of the quality assessment

Authors and year of Publication	Random selection of participants	Definition inclusion/ exclusion criteria	Loss of follow-up	Validity of assessment	Statistical analysis	Reported potential the risk of bias
Kale et al., 2010	No	Yes	yes	Yes	No	high
Kumar et al., 2011	No	yes	yes	yes	No	high
Patter et al., 2014	yes	yes	yes	yes	yes	low
Sudhakar et al., 2015	Yes	yes	yes	yes	yes	low

### Effect of intervention

A summary of the results is presented in Table 3.

**Table 3:** Summary of the intervention effect

	Approach				Transbuccal							Extraoral				

Author/year	Transoral/transbucal	Extraoral	INF%	MO%	PR%	NU/MU%	TR%	SC%	FW%	INF%	MO%	PR%	NU/NU%	TR%	SC%	FW%
Kale et al., 2010	10	4	0	0	_	_	0	0	0	0	50	_	_	0	100	0
Kumar et al., 2011	15	30	0	6.7	0	_	_	_	_	16.6	0	3.3	_	_	_	_
Pattar et al., 2014	8	10	12.5	12.5	0	_	0	0	_	10	40	10	_	0	10	_
Sudhakar et al., 2015	15	15	20	0	_	0	0	VRS 3.6	13.3	20	0	_	0	0	VRS 6.7	53

INF = Infection, MO = Malocclusion, PR = Plate removal, NU/MU = Non union/Malunion, TR = Trismus, SC = Scar, FW = Facial weakness.

### Scar from the aesthetic point of view

Three studies with 62 fractures evaluated scarring. In two of these studies with an extraoral approach the incidence of the scar was 55% (range 10 % to 100% while the transbuccal approach showed no obvious scar. The third study with 30 fractures divided into 15 patients in each group evaluated the scar using the Vancouver scar rating scale. The scar rating scale showed a value of 3.6 with the transbuccal approach and 6.73 in the extraoral approach patients.

### Facial nerve damage evaluation

Two studies with 44 fractures divided into 19 fractures in the extraoral approach and 25 fractures in the transbuccal approach evaluated facial nerve function. The incidence of facial nerve weakness in the transoral group 6.6 % (range, 0 % to 13.3%) and in the extraoral approach the incidence was 26.5% (range, 0 % to 53%).

### Tooth in the line of fracture and its implication on malunion and non-union

The incidence of nonunion was assessed in one study with 30 fractures divided into 15 in the transbuccal group and 15 in the extraoral group. The incidence of non-union in both groups was 0%.

### Infection

A total of 107 fractures enrolled in four studies evaluated the incidence of infection, 48 fractures in the transbuccal approach group and 59 fractures in the extraoral group. In the transbuccal group, the incidence of infection was 8.1% (range, 0% to 20%) whereas in the extraoral group the incidence of infection was 11.7% (range, 0 % to 20%).

### Malocclusion

Four studies with 107 fractures divided into 48 in the transbuccal and 59 in the extraoral group assessed the incidence of malocclusion. In the transbuccal group, the incidence of malocclusion was 4.8% (range, 0% to 12.5%)whereas in the extraoral group the incidence of malocclusion was 22.5% (range, 0% to 50%).

### Mouth opening (trismus)

Three studies analysed the incidence of trismus postoperatively with 62 fractures divided into 33 in the transbuccal group and 29 in the extraoral group. The incidence of trismus was 0% in both groups.

### Plate removal

The incidence of plate removal was reported in two studies with 63 fractures divided into 23 in the transbuccal group and 40 fractures in the extraoral group. The incidence of plate removal in the transbuccal group was 0% whereas the incidence of plate removal in the extraoral group was 6.7% (range, 3.3% to 10%).

## Discussion

The surgical approach in the management of mandibular fractures has been an ongoing point of debate some authors advocating the transbuccal approach and others the extraoral approach still others advocate a combination approach [18]. To the best of our knowledge, there is no systematic literature review comparing the transbuccal and extraoral approaches for mandibular angle fractures.

The extraoral approach provides easy access and direct visualisation, but it is associated with marginal mandibular nerve injury and an often visible scar [12]. In a study by Toma et al. [8] no significant difference in the complication rate was reported between the transoral and extraoral approaches for the treatment of mandibular fractures, including body, angle, and ramus. Angle fractures are more difficult to treat with the transoral approach than anterior mandibular fractures and they have a higher incidence of complications such as infection and non-union.

It has been shown that when the surgeon shifts from the transoral approach to the extraoral approach intra-operatively the complication rate increases, Therefore, a preoperative decision about the surgical approach should be made. The extraoral approach theoretically provides a cleaner wound by separating the sterile skin from the contaminated oral cavity [8]. The extraoral approach also allows direct visualisation of both medial and lateral cortices to assist with proper reduction [19]. Unfortunately, the extraoral route may cause an unsightly scar [8, 12, 17].

The transbuccal approach is usually advocated because it results in no external scar and allows direct visualisation and confirmation of the proper occlusion during placement of the bone plates [12]. Despite the advantages of this approach, it is through a contaminated area that might increase the risk of infection. Transbuccal trocar instrumentation is a sensitive technique and the surgeon has to be familiar with the armamentarium and be skilled in the use of the trocar cannula. In the literature, there is some debate about identifying a safe and accurate technique for transbuccal incisions [20]. It has been suggested that the surgeon’s inexperience will lead to additional facial incisions, especially when access is severely limited due to the nature of the masseteric region, and there is a risk of damaging the facial nerve [21, 22].

From an aesthetic point of view, the extraoral route can cause an obvious unsightly scar. Three studies [9, 10, 12] assessed the incidence of the postoperative scar and two of these studies found that the mean extraoral scar was 55 %, while transbuccal approach showed no obvious unsightly scar. While the remaining study evaluated the scar using the Vancouver scar rating scale which showed a value of 3.6 with the transbuccal approach and 6.73 in the extraoral approach patients. Some authors attributed hypertrophic scar formation to abnormal healing processes. Although the processes leading to hypertrophic scar formation are not yet clarified, altered apoptotic behaviour was believed to be a significant factor [23].

Facial nerve injury is a common complication encountered with the extraoral approach. Two of the included studies assessed facial nerve function postoperatively and found that the incidence of postoperative transient facial nerve weakness in the transbuccal group was 6.6% and in the extraoral approach, the incidence was 26.5%. The complication could be attributed to the blunt trauma caused due to soft tissue retraction and tissue dissection [9].

Nonunion and delayed union usually result from infection or conditions that decrease the blood supply after mandibular fracture treatment [24]. The incidence of nonunion and malunion is between 1% and 2% in the literature [25]. What to do with a tooth in the line of fracture is always a question. Regarding its implication on malunion and non-union, tooth in the line of fracture has been implicated among causes of non-union in mandibular fractures [26]. one of the investigated study [9] assessed nonunion was 0% in both groups.

Infection is the most common complication with mandibular fractures, especially those at the angle. Infections evaluated in all included studies, Infections were 8.1% with the transbuccal approach and 11.7% with the extraoral approach which could be due to increased operative time and improper patient maintenance and wound dehiscence [9]. Some authors claim that infection is attributable to poor oral hygiene, inappropriate post-operative instructions, longer operative time and surgical technique but not the hardware used, others blame fixation hardware [27]. Successful treatment of mandible fractures depends on undisturbed healing in the correct anatomical position under stable conditions. Failure to achieve this leads to infection, malocclusion or nonunion [24]. Some authors claim that the use of a single miniplate leads to more infections than when two- mini plates are employed [28, 29]. However, the process of putting the second miniplate at the lower border means increased periosteal stripping, bacterial contamination and added hardware on the mandible, which theoretically can increase the possibility of infection [17, 25].

Plate removal was much higher in the extraoral approach than with the transoral approach (6.7% versus 0%). The need for plate removal was attributed to infection and wound dehiscence [10]. Four studies reported a 4.8% malocclusion rate with the transbuccal approach and 22.5% with the extraoral approach. In analysing the cause of malocclusion the patients had an associated second fracture on the contralateral side and this may be a confounding factor. In some cases, malocclusion was the result of a sub-optimal reduction at operation or inadequate stability after treatment [24, 25]. Mouth opening (trismus), three studies analysed the incidence of trismus postoperatively. The incidence of trismus was 0% in both groups. Two studies did not mention the duration of surgery. Therefore, an appropriate comparison regarding the mean operation time was not possible. However, the dissection through multiple tissue layers and the closure with the extraoral approach obviously increases the duration of surgery.

The results of this study suggest that transbuccal approach shows fewer complications than extraoral approach when used for the treatment of mandibular angle fractures.
